# Temporal Dynamics of T Cell Immunity Induced by TbpB^Y167A^ Vaccine in Colostrum-Deprived Piglets Challenged with *Glaesserella parasuis*

**DOI:** 10.3390/vetsci13010073

**Published:** 2026-01-11

**Authors:** Alba González-Fernández, María José García-Iglesias, César B. Gutiérrez-Martín, Óscar Mencía-Ares, Sonia Martínez-Martínez

**Affiliations:** 1 Department of Animal Health, Faculty of Veterinary, Universidad de León, 24071 León, Spain; cbgutm@unileon.es (C.B.G.-M.); oscar.mencia@unileon.es (Ó.M.-A.); smarm@unileon.es (S.M.-M.); 2 Instituto de Desarrollo Ganadero y Sanidad Animal (INDEGSAL), Universidad de León, 24071 León, Spain; 3 Instituto de Biomedicina (IBIOMED), Universidad de León, 24071 León, Spain

**Keywords:** cellular immune response, colostrum-deprived piglets, flow cytometry, *Glaesserella parasuis*, intranasal challenge, oral immunization, subunit vaccine, swine, T cell subsets

## Abstract

*Glaesserella parasuis* (*G. parasuis*) is a key pathogen responsible for swine respiratory disease, and the development of broadly protective vaccines is hampered by its high antigenic diversity. The iron-acquisition protein TbpB is a conserved vaccine candidate, but the cellular immune responses it elicits, particularly T-cell subset dynamics during immunization and challenge, remain insufficiently defined. This study characterized these responses after oral immunization of colostrum-deprived piglets with the TbpB^Y167A^ mutant. Ten colostrum-deprived piglets were allocated to immunized and non-immunized (PBS) groups, immunized at days 15 and 30 of life and subsequently challenged with *G. parasuis* (45 days old); peripheral blood mononuclear cells were collected at baseline, after each immunization, and at 1 and 3 days post-infection. Multiparametric flow cytometry was used to quantify major leukocyte subsets and T-cell phenotypes defined by sIgM, CD172a, CD3, TCRγδ, CD8α/β, CD4 and CD27 expression. Booster immunization induced significant expansion of B cells (*p* < 0.01), TCRγδ T cells (*p* < 0.01), CD8^+^ αβ T cells (*p* < 0.001) and CD4^+^ memory T cells (*p* < 0.01) in immunized piglets compared with controls. After challenge, CD8^+^ cytotoxic T cells in immunized animals rapidly shifted from naïve to memory phenotypes, peaking at 48–72 h (*p* < 0.01). These biphasic T-cell dynamics are consistent with the protective efficacy previously demonstrated for this vaccine in colostrum-deprived piglets, and support a key contribution of TCRγδ, CD8^+^ cytotoxic and CD4^+^ memory T cells to immunity against *G. parasuis* and to the design of next-generation vaccines.

## Simple Summary

*Glaesserella parasuis* is an important cause of respiratory and systemic disease in piglets, leading to animal welfare issues and economic losses in pig production. The high antigenic diversity of this bacterium makes it difficult to develop vaccines that provide broad and reliable protection. In this study, colostrum-deprived piglets were vaccinated with a genetically modified form of a bacterial surface protein that is involved in iron acquisition, and later exposed to infection under experimental conditions. Blood samples were collected at different time points to analyze how the immune system responded to vaccination and subsequent infection. Vaccinated animals showed a marked increase in several lymphocyte populations, including cells with cytotoxic activity and cells with a long-lasting response to the pathogen. These changes were observed only after booster vaccination and again shortly after infection, and were associated with protection against disease. Overall, these findings indicate that this vaccine candidate can stimulate a coordinated cellular immune response that may contribute to improved control of *Glaesserella parasuis* infections in pig herds.

## 1. Introduction

The swine industry faces significant economic challenges, with Glässer’s disease being one of the most relevant [1]. This disease is caused by *Glaesserella parasuis* (*G. parasuis*), a pathogen that colonizes the upper respiratory tract of pigs and, under stressful conditions such as weaning or transport, can trigger severe systemic infections [2]. In addition, this bacterium is one of the secondary pathogens included in the porcine respiratory disease complex (PRDC), where it plays a major role in coinfections and contributes to the severity of respiratory disease in pigs [3,4]. Piglets in the early stages of lactation and fattening are particularly vulnerable, resulting in elevated morbidity and mortality rates [5].

Vaccination remains the most effective approach for preventing and controlling Glässer’s disease; however, the lack of a universal commercial vaccine and the high genetic and antigenic variability among strains limit the efficacy of current autogenous vaccines [6]. Thus, the search for effective heterogenous vaccines is an open line of research where some virulence factors have been characterized, but the complete pathogenic profile of *G. parasuis* remains poorly defined. In this context, it has been demonstrated that *G. parasuis* employs virulence mechanisms to establish infection, such as iron acquisition from porcine transferrin, mediated by the surface proteins TbpA and TbpB, which facilitate bacterial survival in iron-restricted environments like mucosal surfaces [7,8,9,10]. In addition, Martínez-Martínez et al. [11] demonstrated that the TbpB protein can elicit both B and T cell responses in pigs, supporting its relevance as a vaccine antigen. Therefore, subunit vaccines, particularly those incorporating targeted mutations of TbpA and TbpB, represent a promising strategy for achieving broader and more consistent protection [7,11,12,13,14,15,16,17].

Cellular immune responses, particularly those mediated by T lymphocytes, are fundamental for host defense against bacterial infections and play an essential role in the development of effective vaccines in swine and other species [18]. T cells recognize pathogens in an antigen-specific manner via their unique surface receptors, and upon activation, proliferate and secrete cytokines that orchestrate a coordinated immune response [18]. In the porcine model, both CD4^+^ helper and CD8^+^ cytotoxic T cell subsets contribute to pathogen clearance and shape the outcome of infection with a range of bacterial agents, including *G. parasuis* [15,19]. Although humoral factors such as antibody production are important, emerging evidence indicates that T cell-mediated immunity is central for controlling *G. parasuis* infection and for achieving vaccine-induced protection [19,20]. However, detailed phenotypic and functional characterization of T cell subpopulations in the context of natural or experimental infection by *G. parasuis* remains scarce. Only a few studies to date have used flow cytometry or similar immunophenotypic approaches to track changes in CD3^+^, CD4^+^, and CD8^+^ T lymphocyte compartments in *G. parasuis*-infected pigs or after immunization with subunit vaccine, revealing that infection can lead to dynamic alterations in these subsets and underlining the need for further studies in this field [15,21]. The immune changes described previously included leukopenia [22], reductions in CD25^+^ (a marker of T cell activation) and CD3^+^ T cells, and increases in αIgM^+^ B cells, monocytes, and granulocytes in *G. parasuis*-infected pigs [21]. However, a deeper understanding of the specific lymphocyte subsets involved in protective immunity remains lacking. Therefore, this study investigates the temporal dynamics of the peripheral blood mononuclear cell (PBMC) subpopulations in colostrum-deprived piglets immunized with a subunit vaccine based on a modified TbpB (mutant Y167A) and subsequently challenged with highly virulent field isolates of *G. parasuis*. The aim is to better define the cellular mechanisms underlying vaccine-induced protection with particular attention to T lymphocyte subpopulations.

## 2. Materials and Methods

### 2.1. Bacterial Isolate, Growth Conditions and Dose Preparation for Experimental Infection

Three highly virulent field isolates of *G. parasuis* (serovars 1, 4 and 7) were used for experimental challenge as part of a broader investigation on protection against Glasser’s disease [17]. All strains were cultured from frozen stocks onto chocolate agar plates (Oxoid, Basingstoke, UK) and incubated at 37 °C for 48 h under microaerophilic conditions. Inoculum preparation followed a standardized protocol: colonies were resuspended in pleuropneumonia-like organism (PPLO) broth (Pronadisa, Madrid, Spain) supplemented with nicotinamide adenine dinucleotide (NAD) (2 µL/mL of 20 mg/mL stock) and glucose (5 µL/mL of 50% *v*/*v* stock) and grown at 37 °C with shaking until mid-log phase (OD_600_ 0.1–0.2). Cultures were then expanded in fresh medium to an optical density (OD_600_) of 0.025–0.03, centrifuged at 1300× *g* for at least 25 min, and resuspended in Roswell Park Memorial Institute (RPMI) 1640 medium (Gibco, Thermo Fisher Scientific, Grand Island, NY, USA). Viable bacterial concentrations (CFU/mL) were determined by serial dilution plating as previously described [17].

### 2.2. Experimental Design

This study involved 10 colostrum-deprived Landrace × Large-White piglets, reared artificially as described by Guizzo et al. [15]. All animals were confirmed free of *G. parasuis* by PCR analysis of nasal swabs prior to the experiment. Piglets were randomly assigned to two groups with five in each group and housed in separate rooms within biosafety level 2 facilities.

The experiment was carried out in two phases, following the protocol described in a previous study [17]. In the first phase, five piglets received oral mucosal immunization with two doses of the TbpB^Y167A^ mutant vaccine [23], administered on days 15 and 30 of age. The remaining animals served as non-immunized controls and were given two administrations of phosphate-buffered saline (PBS) in equivalent volumes. The second phase started on day 45 (15 days after the booster dose), when both groups were intranasally challenged with a field isolate of *G. parasuis* at a final dose of 5 × 10^6^ CFU. This challenge phase lasted 7 days, after which all surviving piglets were euthanized.

All procedures involving animals were reviewed and approved by the Animal Welfare and Ethics Committee of the University of León (OEBA-ULE-016-2023). Piglet handling was performed by licensed veterinarians and trained personnel in compliance with current Spanish and European Union regulations (Royal Decree 53/2013 on the protection of animals used for scientific purposes; Directive 2010/63/EU on the protection of animals used for scientific purposes). Upon arrival, all animals underwent a clinical health evaluation and were closely monitored throughout the study.

### 2.3. Isolation and Cryopreservation of Peripheral Blood Mononuclear Cells

Peripheral blood was aseptically collected from the jugular vein of manually restrained pigs using a vacutainer system. Needle gauges (Becton Dickinson, Franklin Lakes, NJ, USA) of 20 G or 18 G were used depending on the size and age of the animals (20 G needles for younger, smaller piglets and 18 G needles for older, larger pigs) to ensure safe and efficient blood collection. Blood samples were transferred into 4 mL EDTA-coated tubes containing 7.2 mg of K_3_-EDTA (BD Vacutainer^®^, Becton Dickinson, Franklin Lakes, NJ, USA) for PBMC isolation. Samples were collected at five time points corresponding to three experimental periods: (a) baseline, immediately before administration of the first vaccine dose (day 15), to establish profiles of the evaluated immune cell subpopulations without subsequent statistical comparison; (b) post-vaccination, prior to the second immunization (day 30) and after the second immunization but before infection (day 45); and (c) post-infection, including 1 and 3 days post-infection (dpi).

Peripheral blood mononuclear cells (PBMCs) were isolated from anticoagulated blood by density gradient centrifugation using Ficoll-Paque™ (Sigma-Aldrich, St. Louis, MO, USA), following the protocol described in another study [12] with slight modifications. Briefly, 6 mL of Ficoll-Paque™ was carefully layered beneath 3 mL of whole blood in a glass tube (Corning Incorporated, Corning, NY, USA). The samples were centrifuged at room temperature (20 °C) for 35 min at 650× *g* without brake. Mononuclear cells (lymphocytes and monocytes) were collected from the interphase between the Ficoll-Paque™ and the plasma. The PBMCs were then washed twice with 1× phosphate-buffered saline (PBS) supplemented with 2% fetal bovine serum (FBS) (Gibco, Thermo Fisher Scientific, Grand Island, NY, USA)by centrifugation at 450× *g* for 10 min with brake, and the resulting pellet was resuspended in 1.5 mL of cryopreservation medium consisting of 10% dimethyl sulfoxide (DMSO) (ThermoFisher Scientific, USA) and 90% FBS, then transferred to cryovials of 2 mL (Sigma-Aldrich, St. Louis, MO, USA).

For cryopreservation, the cryovials containing resuspended PBMCs were placed into alcohol-filled freezing containers (100% isopropanol) (Sigma-Aldrich, St. Louis, MO, USA) to ensure a controlled cooling rate and reduce cell mortality. These containers were kept at –80 °C for 24 h. After this initial freezing step, the vials were transferred to a liquid nitrogen storage tank for long-term preservation.

### 2.4. Flow Cytometry

#### 2.4.1. Staining and Immune Cell Count

PBMCs were thawed by briefly opening the cryovials to release internal pressure. Cells were immediately transferred to 1 mL of 1× PBS supplemented with 2% fetal calf serum (FCS) or FACS buffer (1× PBS containing 0.1% bovine serum albumin and 0.01% sodium azide), depending on the downstream application. Following centrifugation at 300× *g* for 7 min at 4 °C, the supernatant was discarded, and the cell pellet was resuspended in up to 10 mL of PBS + 2% FCS.

Cell viability and concentration were assessed by Trypan Blue exclusion (1:10 dilution, Sigma-Aldrich, St. Louis, MO, USA), and cell counts were performed individually for each animal. Based on the total yield and required final volume, cell suspensions were adjusted to achieve a concentration of 1 × 10^6^ cells per 200 µL. Subsequently, 200 µL of this suspension was added to each well of V-bottom 96-well plates (Thermo Fisher Scientific, Grand Island, NY, USA), ensuring 1 × 10^6^ cells per well.

After an additional centrifugation step (3 min at 300× *g* and 4 °C), pellets were gently loosened by vortexing and washed twice more in 1× PBS. Viability staining was performed using the LIVE/DEAD™ Fixable Aqua Dead Cell Stain Kit (Invitrogen, Thermo Fisher Scientific, Grand Island, NY, USA). The dye was diluted in dimethyl-sulfoxide (DMSO), and 200 µL of the working solution (PBS + Live/Dead reagent) was added per well, followed by incubation for 30 min at 4 °C in the dark.

Blocking was carried out using FACS buffer supplemented with 5% goat serum and 5% porcine serum (50 µL/well), except when surface immunoglobulins were to be detected, in which case porcine serum was omitted to avoid cross-reactivity. After 15 min of incubation at 4 °C, cells were washed twice with FACS buffer.

Primary monoclonal antibodies or hybridoma supernatants were prepared according to the experimental design and added to each well, followed by 30 min of incubation at 4 °C. After washing, secondary antibodies were diluted in FACS buffer containing 5% porcine serum and added to each well (50 µL/well), followed by a further 30 min incubation at 4 °C. A second blocking step using 10% normal mouse serum in FACS buffer (50 µL/well) was performed for 15 min at 4 °C prior to streptavidin addition, followed by three washes.

Finally, cells were resuspended in 200 µL of FACS buffer and transferred to pre-labeled cytometry tubes (Corning Incorporated, Corning, NY, USA) containing 300 µL of fixation buffer (0.1% paraformaldehyde in FACS buffer), unless immediate acquisition was planned, in which case fixation was omitted. A total of 80,000 events were acquired in a FACSCanto II cytometer (Becton Dickinson, Franklin Lakes, NJ, USA), and data were analyzed with FlowJo software v10.10.0 (Becton Dickinson, Franklin Lakes, NJ, USA). Lymphocyte populations were identified based on their distribution in forward scatter (FSC) versus side scatter (SSC) dot plots. The analysis was performed using absolute values, calculated for each subpopulation by multiplying the total number of acquired events by the percentages obtained from sequential gating.

#### 2.4.2. Immunostaining Panels

All immunostaining panels used throughout the present study are detailed in Table 1.

Primary monoclonal antibodies were hybridoma supernatants generously donated by the Porcine Immunology Laboratory of INIA (Spain), except for clones PGB22A (Kingfisher Biotech, Saint Paul, MN, USA) and PG164A (Kingfisher Biotech, Saint Paul, MN, USA). Secondary antibodies were commercial antibodies obtained from Southern Biotech (SB) (Birmingham, AL, USA) or BD-Biosciences (BDB) (San Jose, CA, USA) and they are conjugated to phycoerythrin (PE), phycoerythrin-Cyanine 7 (PECy7), allophycocyanine (APC) or streptavidin-BrilliantViolet421 (StrAv-BV421).

These panels were specifically chosen as they are widely used in vaccine response studies to characterize key porcine PBMC subsets, focusing on the immune response to immunization rather than differentiating between health and disease states [24,25]. These panels were designed to comprehensively characterize porcine PBMCs at key experimental time points, including pre-vaccination, post-vaccination, and post-infection. The first set of panels allowed identification of major leukocyte subsets, including T, B, NK, and myeloid cells (monocytes), whereas additional panels focused on T cell subpopulations, distinguishing naïve, memory, and cytotoxic subsets.

Combined panels applied after bacterial challenge integrated these markers to enable broader profiling of both major leukocyte populations and T cell differentiation or activation states. Appropriate controls were included in all experiments to ensure accurate gating and assessment of non-specific staining. These comprised fluorescence-minus-one (FMO), unstained, and isotype-matched samples. Irrelevant hybridoma supernatants used as isotype controls included 4B9 (IgG1), 1E12 (IgG2a), 3E4 (IgG2b), and 3E6 (IgG1, biotin-conjugated), matched by isotype to the primary antibodies in each panel. All hybridoma supernatants (primary monoclonal antibodies) were generously donated by the Porcine Immunology Laboratory of INIA (Madrid, Spain), except for clone PGB22A (Kingfisher Biotech, Saint Paul, MN, USA) and PG164A (Kingfisher Biotech, Saint Paul, MN, USA).

All antibodies from the panels were titrated prior to the study to determine optimal working concentrations. Serial dilutions were prepared in FACS buffer and tested using PBMCs from a healthy adult pig. The dilution selected for use in the study was the highest concentration that did not produce significant alterations in population frequencies or staining intensities, ensuring signal specificity and minimizing background fluorescence while maintaining clear immunophenotypic resolution.

The lymphocyte subpopulations analyzed and their phenotypic characterization are shown in Table 2.

### 2.5. Statistical Analysis

Statistical analyses were performed using SPSS software version 29.0 (IBM, SPSS Statistics, Armonk, NY, USA). Data normality was assessed using the Shapiro–Wilk test. To compare immunized and non-immunized animals, independent-samples *t*-tests were conducted for each staining panel after checking normality (Shapiro–Wilk) and homogeneity of variances (Levene’s test). Population dynamics over time after the first and second vaccine doses were evaluated using a generalized linear model (GLM) with estimated marginal means, including five distinct T cell subpopulations and immunization status (immunized vs. non-immunized) as a fixed factor (see Table 2 for details of the analyzed T cell subpopulations and their markers). Across these analyses, multiple testing arising from the evaluation of several cell populations and time points was addressed by applying Bonferroni correction to all pairwise comparisons within each panel or model to control for type I error. Following infection, paired-samples *t*-tests were performed to analyze changes in T cell subpopulations within the same animals at 1 and 3 dpi. Due to the limited number of non-immunized animals at 3 dpi (*n* = 2), statistical analysis at these time points was restricted to immunized animals. A *p*-value < 0.05 was considered statistically significant while *p*-values < 0.1 were considered as a trend. Graphs and figures were generated using GraphPad Prism version 10.6.0 (GraphPad Software, San Diego, CA, USA) and SPSS software, and flow cytometry plots were created using FlowJo version 10.9.0 (BD Biosciences, San Jose, CA, USA).

## 3. Results

### 3.1. Response of Peripheral Blood Mononuclear Immune Cells to Immunization

**Figure 1:**
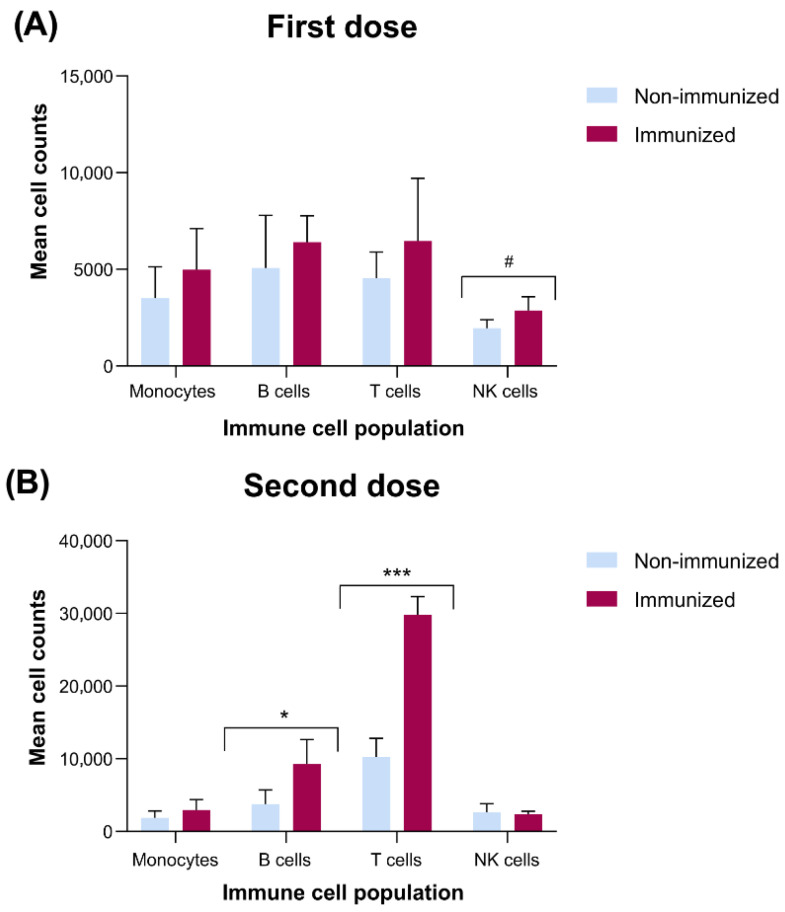
Mean cell counts of PBMC subsets in immunized and non-immunized piglets after the first (**A**) and second (**B**) vaccine dose. Bars represent mean + standard deviation (SD) of monocytes (CD3^−^ sIgM^−^ CD8α^−^ CD172a^+^), B cells (CD3^−^ sIgM^+^ CD8α^−^ CD172a^−^), T cells (CD3^+^ sIgM^−^ CD8α^+^/^−^ CD172a^−^), and NK cells (CD3^−^ sIgM^−^ CD8α^+^ CD172a^−^). Independent-samples *t*-test was applied for statistical analysis. *p* < 0.05 (*), *p* < 0.001 (***); #, trend (*p* < 0.1).

Analysis of PBMCs following the administration of the first vaccine dose revealed no statistically significant differences in monocyte, B cell or T cell populations between immunized and non-immunized pigs (Figure 1A). Thus, the response to a single dose of the vaccine only showed a trend towards significance in the number of NK cells represented by a reduced count in the non-immunized pigs compared to those immunized (*p* < 0.1).

Interestingly, the results obtained on day 45 (after the second vaccine dose) revealed more noteworthy changes in immune cell composition. Immunized piglets exhibited significantly higher B cell counts (*p* < 0.05) and a markedly increased number of total T cells (*p* < 0.001) compared to non-immunized ones (Figure 1B). However, no significant differences were detected in monocyte or NK cell counts associated with the immunization with two doses.

### 3.2. T Cell Subpopulations Dynamics Following Immunization

To assess the immunological impact of immunization, five T cell subpopulations (TCRγδ, CD8^+^, CD4^+^ naïve and memory cells and double-negative αβ cells) were quantified in samples from immunized and non-immunized animals (Table 2).

**Figure 2:**
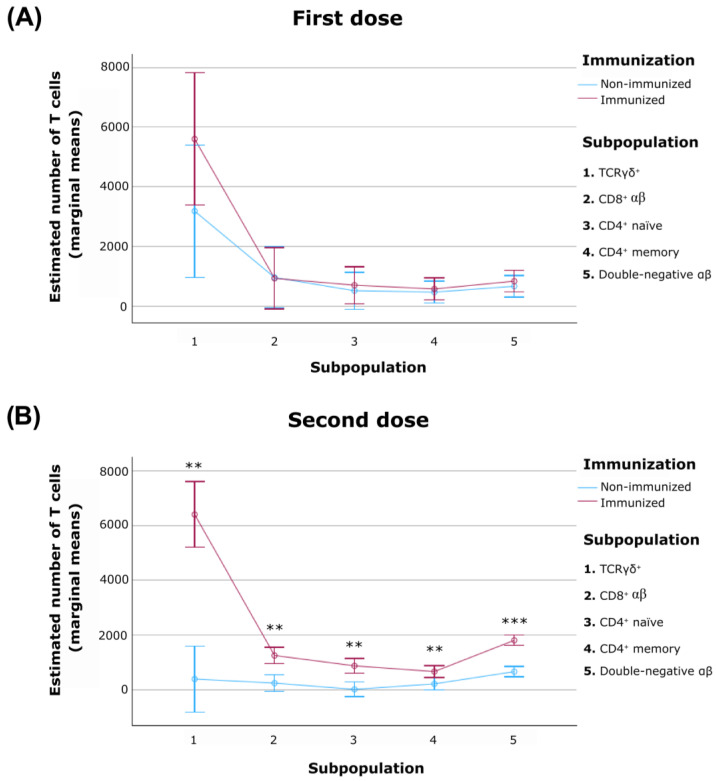
Dynamics of T cell subpopulations in immunized and non-immunized pigs following the first (**A**) and second (**B**) vaccine doses. Absolute numbers of five T cell subsets were analyzed by flow cytometry: TCRγδ cells (CD3^+^ TCRγδ^+^), CD8^+^ αβ cells (CD3^+^ TCRγδ^−^ CD8β^+^), CD4^+^ naïve cells (CD4^+^ CD27^+^ CD8α^−^), CD4^+^ memory cells (CD4^+^ CD27^−^ CD8α^+^) and double-negative αβ cells (CD3^+^ TCRγδ^−^ CD4^−^ CD8β^−^). Data are shown as means ± standard error of the mean (SEM) for each group at the indicated time points (after the first and second vaccine doses). *p* < 0.01 (**), *p* < 0.001 (***).

The results obtained indicated that administration of a single dose did not induce statistically significant differences associated with immunization in any of the five evaluated subpopulations (Figure 2A). However, after the second dose, immunized animals exhibited significant increases across both major T cell compartments (TCRγδ and αβ) (Figure 2B). Specifically, both TCRγδ cells (CD3^+^ TCRγδ^+^) (*p* < 0.01) and CD3^+^ TCRγδ^−^ cells (*p* < 0.01) showed notable expansion, with the CD8^+^ αβ cells subset demonstrating a particularly marked increase (*p* < 0.01). Within the CD4^+^ αβ compartment, both naïve (*p* < 0.01) and memory (*p* < 0.01) subsets also expanded substantially. Among non-conventional populations, double-negative αβ cells were also significantly elevated (*p* < 0.001).

### 3.3. Early Immune Response to Immunization After Infection

**Figure 3:**
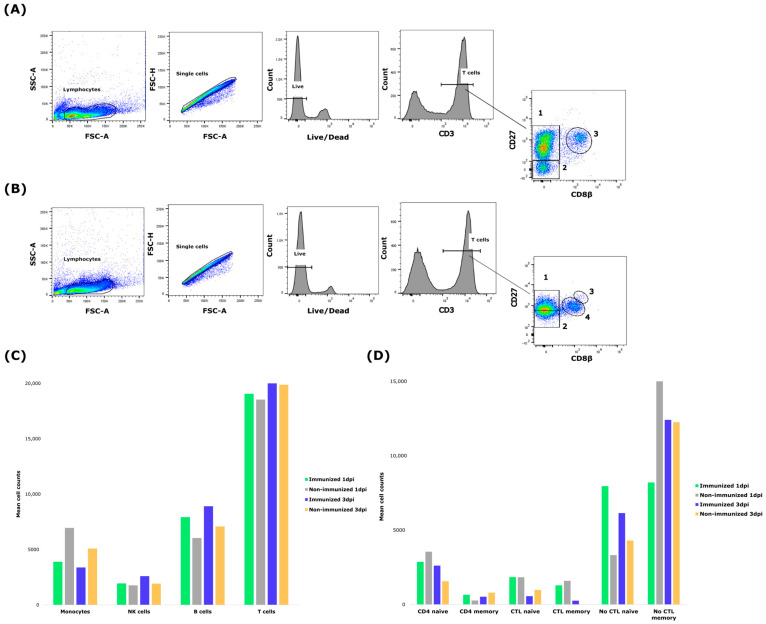
Post-infection PBMC and T-cell subset analysis. (**A**,**B**) Representative gating strategy for the analysis of CD8^+^ T-cell subpopulations in immunized piglets at 1 and 3 days post-infection (dpi), respectively. Lymphocytes were selected by FSC/SSC profile, followed by single-cell and live-cell discrimination; CD3^+^ T cells were subsequently analyzed based on CD27 and CD8β expression. Subpopulations are indicated by numbers: 1 = naïve non-CTL (CD3^+^ CD8α^+^ CD8β^−^ CD27^+^), 2 = memory non-CTL (CD3^+^ CD8α^+^ CD8β^−^ CD27^−^), 3 = naïve CTL (CD8β^+^ CD27^+^) and 4 = memory CTL (CD8β^+^ CD27^−^). (**C**) Mean cell counts of major PBMC subsets (monocytes, NK cells, B cells and T cells) in immunized and non-immunized pigs at 1 and 3 dpi. Bars represent group means (*n* = 5 at 1 dpi; *n* = 5 immunized and *n* = 2 non-immunized at 3 dpi); no significant differences were detected between immunized and non-immunized animals at either time point. (**D**) Mean cell counts of CD4^+^ naïve, CD4^+^ memory, naïve and memory CTL, and naïve and memory non-CTL T-cell subpopulations in immunized and non-immunized pigs at 1 and 3 dpi. Because only two non-immunized pigs remained at 3 dpi, data for this group are shown descriptively and statistical analyses were restricted to paired comparisons within the immunized animals.

In the post-infection period, no significant differences were detected in the counts of major PBMC subsets (monocytes, B cells, T cells, and NK cells) between immunized and non-immunized animals at either 1 or 3 dpi (Figure 3C). However, at 3 dpi, only two non-immunized pigs remained, so data for this group are shown descriptively and statistical analyses were restricted to paired comparisons within the immunized animals (Figure 3D). Paired comparisons within immunized pigs of the six T cell subpopulations revealed marked changes in the CD8^+^ compartment. Specifically, naïve CTL cells significantly decreased from day 1 to day 3 post-infection (*p* < 0.05), while memory CTL cells significantly increased over the same period (*p* < 0.05) (Figure 3A,B), indicating a rapid shift from a naïve to a memory phenotype. In contrast, CD4^+^ subsets (naïve and memory) remained stable, and while a trend toward decrease was noted in naïve non-CTL T cells (*p* < 0.1), no significant changes were observed in the non-CTL memory subset.

## 4. Discussion

*G. parasuis* remains a significant and complex pathogen in swine medicine, causing severe polyserositis, arthritis, and meningitis, especially in young pigs. Its genetic diversity and variable virulence result in frequent outbreaks even in well-managed herds [1,26,27]. Conventional bacterins have limited efficacy due to suboptimal cross-protection and rapid strain evolution, driving efforts to develop recombinant vaccines targeting conserved antigens such as TbpB, capable of eliciting both humoral and cellular immunity [6,15,17,20,28,29]. However, the complex interplay between innate and adaptive immune responses in pigs, characterized by a high proportion of unconventional T cell subsets and unique lymphocyte ontogeny, necessitates a deeper understanding of vaccine-induced cellular dynamics to optimize protection [30,31].This study addresses the knowledge gap about vaccine-induced immune cellular dynamics by providing a detailed temporal analysis of cellular immunity elicited by a recombinant TbpB-based vaccine, advancing our understanding of progressive lymphocyte activation essential for sustained protection against *G. parasuis* infection. Building upon the framework proposed by Frandoloso et al. [28], who first emphasized the immunological relevance of conserved antigens and the limitations of conventional bacterins, our findings provide functional evidence of coordinated lymphocyte activation supporting lasting immunity against this bacterium. The protective efficacy of the TbpB^Y167A^ vaccine, in terms of survival, clinical scores and lesion severity after *G. parasuis* challenge, has been reported in detail for this vaccine in the same colostrum-deprived piglet challenge model [17], and the present work specifically expands on the cellular correlates of that protection.

In the initial phase of our study, the absence of statistically significant differences in the general immune cell subpopulations associated with the administration of the first TbpB^Y167A^ vaccine dose, prior to infection, does not necessarily indicate a lack of initial immunological priming, but rather reflects the natural kinetics of immune priming, where phenotypic changes in lymphocyte populations manifest progressively after initial sensitization, as described in other porcine vaccine studies. Thus, Zhang et al. [32] demonstrated that immunization against *Actinobacillus pleuropneumoniae* (a Gram-negative respiratory pathogen similar to *G. parasuis* in terms of disease presentation and immunological mechanisms in swine) with a combinatorial bacterin vaccine induced CD4^+^ T cell responses, but these responses were significantly amplified following booster immunization rather than after the primary vaccine dose, confirming that substantial alterations in T cell phenotype generally require additional antigen exposure in the porcine model. Likewise, the trend toward reduced NK cell frequency in non-immunized animals after the inoculation of the first dose of the TbpB^Y167A^ vaccine may suggest early modulation of the innate immune compartment, potentially reflecting the initial engagement of the immune system by the vaccine. Similar transient reductions in systemic NK cell populations have been documented following bacterial immunization in pigs. In this sense, Bilhare et al. reported a significant decrease in NK cell frequencies at day 7 post-live attenuated *Salmonella typhimurium* immunization, which rebounded by day 14, illustrating early but progressive innate activation [33].

An interesting finding was the markedly different immune response following the second TbpB^Y167A^ vaccine dose, with more pronounced changes in the immune cell composition. Thus, the significantly higher B cell counts in the immunized piglets indicate an effective activation of the humoral component of the adaptive immune response. Expansion of B cells post-vaccination is a well-established biomarker of vaccine efficacy, as these cells are responsible for antibody production and long-term protection against bacterial pathogens [34]. In the context of vaccines targeting extracellular bacteria such as *G. parasuis*, the humoral response is essential for generating neutralizing antibodies that can prevent colonization and systemic spread [35]. The findings of the present study are consistent with previous research showing that successful immunization against other pathogens in pigs with two doses leads to a marked increase in B cell populations and antibody titers, correlating with protection against the disease [24,36]. Furthermore, the markedly increased number of total T cells in immunized animals compared to non-immunized ones reflects a cellular basis for long-term immunological memory. It should be noted that the T cell expansion post-vaccination is associated with the development of antigen-specific memory populations capable of rapid and effective responses upon subsequent exposure to the pathogen. Studies in pigs have demonstrated that robust T cell responses, including both helper T cells (CD4^+^, which coordinate immune responses and support antibody production) and cytotoxic T cells (CD8^+^, which directly eliminate infected cells), are essential for optimal vaccine-induced protection, especially against bacterial infections, where cellular immunity complements humoral mechanisms [15,18,19,30]. The expansion of T cells in our study aligns with the expected kinetics of adaptive immune maturation following booster immunization, supporting the establishment of long-term protective immunity.

Based on our results, the T-cell response elicited by the primary and booster doses revealed a well-defined trajectory of adaptive immune maturation. Following the initial immunization, the comparable frequencies of TCRγδ cells, CD8^+^ αβ T cells, naïve and memory CD4^+^ subsets, and double-negative αβ T-cell populations between vaccinated and control animals suggest the establishment of an early priming phase rather than the absence of immunological activation. In contrast, the booster dose induced a marked expansion across all assessed T-cell compartments, with a particularly pronounced increase in TCRγδ T cells. This selective amplification highlights their intermediary function in integrating innate and adaptive immune pathways, consistent with previous findings in pigs challenged with extracellular bacterial pathogens such as *A. pleuropneumoniae* [32]. In the context of *G. parasuis*, the antigenic complexity and immune-evasion strategies described by Frandoloso et al. [28] likely shape these cellular dynamics, reinforcing the relevance of conserved antigens such as TbpB as targets for next-generation vaccines. Recent evidence further indicates that porcine TCRγδ T cells undergo preferential expansion upon secondary antigenic stimulation and exhibit potent cytotoxic activity characterized by the upregulation of canonical effector molecules [37]. These cytolytic properties enable γδ T cells to directly recognize and eliminate stressed or infected host cells, thereby supporting their distinctive role at the interface of innate defense mechanisms and the development of adaptive immunological memory. Moreover, cross-species comparative analyses corroborate that this dual functionality (rapid recall expansion coupled with strong cytotoxic capacity) constitutes a defining feature of γδ T cells in effective vaccine-induced and infection-driven immunity [38].

Concurrently, the pronounced increase in cytotoxic CD8^+^ αβ T cells aligns with evidence that these effector populations undergo robust clonal expansion following booster immunization, thereby enhancing pathogen clearance capacity [24]. Within the CD4^+^ compartment, the expansion of naïve and memory-phenotype subsets is consistent with swine vaccination studies showing that balanced CD4^+^ T cell memory responses support long-term helper function for both humoral and cellular immunity [39]. Finally, the recruitment of double-negative αβ and γδ T cells reveals the contribution of nonconventional T lineages to the vaccine-induced cellular repertoire, suggesting an additional layer of immunological breadth that may strengthen protection against diverse pathogens, consistent with studies documenting proliferative and cytotoxic functions of γδ T cells in vaccine-mediated protection and in porcine models [37,38]. To the best of our knowledge, detailed phenotypic and functional characterization of double-negative αβ T cells in pigs is still very limited, particularly in the context of vaccination or protection against extracellular bacterial pathogens [40]. Nevertheless, the expansion of this subset after booster immunization is compatible with roles described for double-negative T cells in other species, where they can exert regulatory or unconventional effector functions, suggesting that porcine double-negative αβ T cells might contribute to the fine-tuning and breadth of the vaccine-induced response, a possibility that warrants dedicated functional studies. From a practical perspective, the biphasic expansion of memory-phenotype CD8^+^ and CD4^+^ T cells, together with the recruitment of γδ and double-negative αβ T cells, suggests that successful control of Glässer’s disease will require vaccines capable of inducing robust and durable T-cell responses in addition to antibodies. Vaccine platforms and adjuvants that enhance mucosal priming, promote cytotoxic CD8^+^ and γδ T-cell responses, and support the development of long-lived CD4^+^ memory cells may therefore be particularly advantageous for future *G. parasuis* vaccines. Our findings provide a cellular framework that can help guide the selection of adjuvants and delivery routes to preferentially drive these T-cell signatures. However, a limitation of the present study is that we did not directly assess effector functions such as cytokine production or cytotoxic activity of the expanding CD8^+^ αβ and TCRγδ T-cell subsets; thus, their contribution to bacterial clearance and protection is inferred from phenotype and kinetics rather than demonstrated experimentally.

The early immune response dynamics observed in our study align with established patterns of host activation during bacterial infections in swine. The absence of marked differences in circulating lymphocyte subsets at 1 and 3 dpi is compatible with the temporal kinetics of adaptive responses, in which transcriptional and functional reprogramming in blood precede overt phenotypic shifts in immune cell populations, as shown in transcriptomic studies of pigs experimentally infected with *Salmonella enterica* serovar Typhimurium [41]. The rapid conversion from naïve to memory phenotype within the CD8^+^ CTL compartment between 1 and 3 dpi is consistent with previous findings in swine models, where CD8^+^ T cells undergo accelerated differentiation to effector and memory states within days of antigenic stimulation, as demonstrated after immunization and challenge with Classical Swine Fever virus (CSFV) [24]. This phenomenon, described in experimental *G. parasuis* infections in pigs, reflects the capacity of previously primed CD8^+^ T cells to rapidly acquire memory properties and exert recall responses following booster immunization, as evidenced by functional T-cell memory and efficient bacterial clearance post-challenge [39]. The compartment-specific nature of these changes, with CD8^+^ populations showing rapid phenotypic and functional alterations and CD4^+^ subsets remaining comparatively stable, agrees with previous observations in swine models where cytotoxic T lymphocytes display preferential activation and expansion during early challenge. However, CD4^+^ helper subsets maintain stability due to their distinct activation requirements and kinetics, as demonstrated after immunization and virulent CSFV infection [24]. Despite these consistent patterns, our interpretation of early post-challenge dynamics is constrained by the small group size and the reduced number of surviving non-immunized pigs at 3 dpi. Consequently, post-infection findings should be considered descriptive and primarily supportive of the more robust differences detected after booster vaccination.

## 5. Conclusions

Taken together with recent evidence underscoring the biological relevance of distinct T-cell subsets as correlates of protective immunity, our findings underscore the value of flow cytometry as a robust analytical approach for dissecting the complexity of the porcine T-lymphocyte compartment. Our integrative cytometric assessment reveals a well-defined biphasic pattern of cellular immune activation following recombinant TbpB vaccination, characterized by an initial priming phase after the first dose and a pronounced expansion of multiple lymphocyte subsets upon booster administration. The rapid transition from naïve to memory CD8^+^ cytotoxic T cells within 48–72 h post-infection reflects a heightened state of vaccine-induced immune readiness and further substantiates the functional relevance of conserved TbpB antigens. These coordinated cellular dynamics, encompassing both conventional and unconventional T-cell lineages, strongly support the notion that the protective efficacy and cross-reactive immunity observed against heterologous *G. parasuis* strains arise from this accelerated and diversified adaptive response.

Future studies integrating longitudinal cellular profiling with transcriptomic and molecular analyses in relevant target tissues will be essential to elucidate the underlying mechanisms driving these immune responses. Such insights are expected to inform the rational design of next-generation vaccines, ultimately enabling broader and more durable protection against Glässer’s disease.

## Figures and Tables

**Table 1:** Immunostaining panels used in this study.

Primary Murine Monoclonal Antibody	Specificity	Clone	Secondary Antibody Specificity	Fluorochrome (Dilution, Source)
**Panel I:** Major leukocyte subsets, prior to experimental infection, first and second vaccine doses				
sIgM	IgG1	5C9	IgG1	PE (1/100, SB)
CD8α	IgG2a	76-2-11	IgG2a	PECy7 (1/200, SB)
CD172a	IgG2b	74-22-15a	IgG2b	APC (1/2000, SB)
CD3	IgG2b-biotin	BB23-8E6	IgG2b	StrAv-BV421 (1/300, BDB)
**Panel II:** T cell subsets, prior to experimental infection, first and second vaccine doses				
**First combination**				
γδTCR	IgG1	PGB22A	IgG1	PE (1/100, SB)
CD8α	IgG2a	76-2-11	IgG2a	PECy7 (1/200, SB)
CD4	IgG2b	74-12-4	IgG2b	APC (1/2000, SB)
CD3	IgG2b-biotin	BB23-8E6	IgG2b	StrAv-BV421 (1/300, BDB)
**Second combination**				
γδTCR	IgG1	PGB22A	IgG1	PE (1/100, SB)
CD8β	IgG2a	PG164A	IgG2a	PECy7 (1/200, SB)
CD4	IgG2b	74-12-4	IgG2b	APC (1/2000, SB)
CD3	IgG2b-biotin	BB23-8E6	IgG2b	StrAv-BV421 (1/300, BDB)
**Third combination**				
CD27	IgG1	b30c7	IgG1	PE (1/100, SB)
CD8α	IgG2a	76-2-11	IgG2a	PECy7 (1/200, SB)
CD4	IgG2b	74-12-4	IgG2b	APC (1/2000, SB)
CD3	IgG2b-biotin	BB23-8E6	IgG2b	StrAv-BV421 (1/300, BDB)
**Panel III:** Major leukocyte subsets and T cell subsets, day 1 and 3 post-infection				
**First combination**				
sIgM	IgG1	5C9	IgG1	PE (1/100, SB)
CD8α	IgG2a	76-2-11	IgG2a	PECy7 (1/200, SB)
CD172a	IgG2b	74-22-15a	IgG2b	APC (1/2000, SB)
CD3	IgG2b-biotin	BB23-8E6	IgG2b	StrAv-BV421 (1/300, BDB)
**Second combination**				
CD27	IgG1	b30c7	IgG1	PE (1/100, SB)
CD8α	IgG2a	76-2-11	IgG2a	PECy7 (1/200, SB)
CD4	IgG2b	74-12-4	IgG2b	APC (1/2000, SB)
CD3	IgG2b-biotin	BB23-8E6	IgG2b	StrAv-BV421 (1/300, BDB)
**Third combination**				
CD27	IgG1	b30c7	IgG1	PE (1/100, SB)
CD8β	IgG2a	PG164A	IgG2a	PECy7 (1/200, SB)
CD4	IgG2b	74-12-4	IgG2b	APC (1/2000, SB)
CD3	IgG2b-biotin	BB23-8E6	IgG2b	StrAv-BV421 (1/300, BDB)

**Table 2:** Characterization of lymphocyte subpopulations analyzed at different experimental time points.

Panel	Time Points	Subpopulation Description	Lymphocyte Subpopulation (Phenotype Marker)
**I**	Pre-infection, post first and second vaccine doses	Major leukocyte subsets	**Monocytes** (CD3^−^ sIgM^−^ CD8α^−^ CD172a^+^) **B cells** (CD3^−^ sIgM^+^ CD8α^−^ CD172a^−^) **T cells** (CD3^+^ sIgM^−^ CD8α^+^/^−^ CD172a^−^) **NK cells** (CD3^−^ sIgM^−^ CD8α^+^ CD172a^−^)
**II**	Pre-infection, post first and second vaccine doses	T cell subsets	**TCRγδ** (CD3^+^ TCRγδ^+^) **CD8^+^** αβ (CD3^+^ TCRγδ^−^ CD8β^+^) **CD4^+^ naïve** (CD4^+^ CD27^+^ CD8α^−^) **CD4^+^ memory** (CD4^+^ CD27^−^ CD8α^+^) **Double-negative αβ** (CD3^+^ TCRγδ^−^ CD4^−^ CD8β^−^)
**III**	1 and 3 dpi	Major leukocyte subsets and T cell subsets	**Monocytes** (CD3^−^ sIgM^−^ CD8α^−^ CD172a^+^) **B cells** (CD3^−^ sIgM^+^ CD8α^−^ CD172a^−^) **T cells** (CD3^+^ sIgM^−^ CD8α^+^/^−^ CD172a^−^) **NK cells** (CD3^−^ sIgM^−^ CD8α^+^ CD172a^−^) **CD4^+^ naïve** (CD4^+^ CD27^+^ CD8α^−^) **CD4^+^ memory** (CD4^+^ CD27^−^ CD8α^+^) **Naïve CTL** (CD8β^+^ CD27^+^) **Memory CTL** (CD8β^+^ CD27^−^) **Naïve non-CTL** (CD3^+^ CD8α^+^ CD8β^−^ CD27^+^) **Memory non-CTL** (CD3^+^ CD8α^+^ CD8β^−^ CD27^−^)

dpi: days post-infection; NK: Natural Killer; CTL: cytotoxic T lymphocytes; sIgM: surface immunoglobulin M. Phenotype markers represent combinations of surface markers used for immunophenotyping by flow cytometry. Panels correspond to different stages and immunostainings used to analyze the dynamic immune response across the experiment timeline.

## Data Availability

The original contributions presented in this study are included in the article. Further inquiries can be directed to the corresponding author.

## Abbreviations

The following abbreviations are used in this manuscript:

APC	allophycocyanin
CD	cluster of differentiation
CFU	colony-forming units
CTL	cytotoxic T lymphocytes
DMSO	dimethyl sulfoxide
Dpi	days post-infection
FBS	fetal bovine serum
FMO	fluorescence minus one
FSC	forward scatter
GLM	generalized linear model
NAD	nicotinamide adenine dinucleotide
NK	natural killer
OD	optical density
PBMCs	peripheral blood mononuclear cells
PBS	phosphate-buffered saline
PE	phycoerythrin
PECy7	phycoerythrin-cyanine 7
PPLO	pleuropneumonia-like organisms
RPMI	Roswell Park Memorial Institute medium
SIgM	surface immunoglobulin M
SSC	side scatter
StrAv-BV421	streptavidin-brilliant violet 421
TbpB	transferrin-binding protein B
TCR	T cell receptor
